# Supplementary material for: A high-resolution mRNA expression time course of embryonic development in zebrafish
Source: eLife. 2017 Nov 16;6:e30860. doi: 10.7554/eLife.30860 (PMC5690287; doi:10.7554/eLife.30860)
Supplement: Supplementary file 6. [file elife-30860-supp6.zip › biolayout-clusters-files/Cluster175-genes.html]

Cluster175


# Cluster175: Genes

| Ensembl ID | Gene Name | Chr | Start | End | Biotype |
| --- | --- | --- | --- | --- | --- |
| ENSDARG00000029463 | asap1a | 2 | 32281080 | 32372999 | protein\_coding |
| ENSDARG00000052368 | cltb | 14 | 45869575 | 45887900 | protein\_coding |
| ENSDARG00000006758 | fam234b | 3 | 1422721 | 1435692 | protein\_coding |
| ENSDARG00000077002 | igsf3 | 9 | 20265687 | 20562108 | protein\_coding |
| ENSDARG00000020708 | mdkb | 25 | 7748043 | 7756786 | protein\_coding |
| ENSDARG00000014137 | picalmb | 15 | 19791260 | 19836640 | protein\_coding |
| ENSDARG00000034117 | rarga | 23 | 35748397 | 35864932 | protein\_coding |
| ENSDARG00000100109 | trappc2l | 7 | 55332070 | 55335959 | protein\_coding |
